# Supplementary material for: Quantifying Conformational Isomerism in Chain Molecules by Linear Raman Spectroscopy: The Case of Methyl Esters
Source: Molecules. 2021 Jul 27;26(15):4523. doi: 10.3390/molecules26154523 (PMC8348275; doi:10.3390/molecules26154523)
Supplement: Supplementary file 1 [file molecules-26-04523-s001.zip › molecules-1301743-supplementary.pdf]

Supporting Information: Quantifying Conformational Isomerism in Chain Molecules by Linear Raman Spectroscopy: The Case of Methyl Esters

Maxim Gawrilow and Martin A. Suhm*

Institut für Physikalische Chemie, Georg-August-Universität Göttingen, Tammannstr. 6, 37077 Göttingen, Germany

E-mail: msuhm@gwdg.de

Contents

1 Additional Experimental Results **S-3**

1.1 Methyl Methanoate S-3

1.2 Methyl Butanoate S-6

2 Additional Computational Results **S-6**

2.1 Methyl Methanoate S-6

2.2 Methyl Butanoate S-10

3 Instrumental Details **S-13**

3.1 Characteristics of the CCD Chip S-13

3.2 Integration Over the Solid Angle of Observation S-14

4 Explicit Rotational Contours **S-18**

4.1 Symmetric Top S-18

4.2 Separation of γ S-19

4.3 Rotational Temperature S-21

4.4 Rotational Constants S-21

5 Transformation of Raman Results from Turbomole **S-22**

5.1 Obtaining Intensities Comparable to our Setup S-22

5.2 Components of Transition Polarisability S-23

References **S-24**

1 Additional Experimental Results

1.1 Methyl Methanoate

Tab. S1: Integrated intensities I (normalised to ν_{14}) and their relative uncertainties δI of methyl methanoate from a gas phase spectrum. $\tilde{\nu}_{\mathrm{min/max}}$ are the ranges of integration. Errors are estimated as the integral of a linear baseline between start and end point.

Mode	$\tilde{\nu}_{\mathrm{min}}$	$\tilde{\nu}_{\mathrm{max}}$	I	δI/%
$2\nu_{18}$	225	240	2	85
$\nu_{17/16}$	200	460	85	13
$2\nu_{16}$	570	680	2	35
ν_{15}	700	830	12	14
ν_{14}	830	990	100	8
ν_{13}	990	1095	7	69
$\nu_{12/11}$	1095	1195	10	66
ν_{10}	1195	1280	8	65
ν_{9}	1280	1400	23	46
$\nu_{8/7/6}$	1400	1670	37	65
ν_{5}	1670	1820	45	8

Tab. S2: Integrated relative signal intensities from spectra with varying ester concentration (lowest five traces in Fig. 3 in main part). The spectrum with a relative concentration of 2.4 (bold trace) was recorded twice on different days. Intensities are normalised to give 100 in sum, uncertainties are estimated from the noise level.

Range/cm^{-1}	10.2	5.1	2.4 (I)	2.4 (II)	1	1, higher T
1340–1400	18.5 ± 0.4	18.6 ± 0.4	18.9 ± 0.4	18.7 ± 0.5	18.7 ± 0.6	18.1 ± 0.5
1420–1520	31.8 ± 0.5	31.7 ± 0.4	31.3 ± 0.5	31.7 ± 0.6	31.4 ± 0.7	31.2 ± 0.7
1560–1630	2.3 ± 0.4	2.5 ± 0.4	2.5 ± 0.4	2.6 ± 0.4	2.8 ± 0.6	2.4 ± 0.5
1710–1780	47.4 ± 0.4	47.2 ± 0.4	47.2 ± 0.5	47.0 ± 0.5	47.0 ± 0.7	48.3 ± 0.7

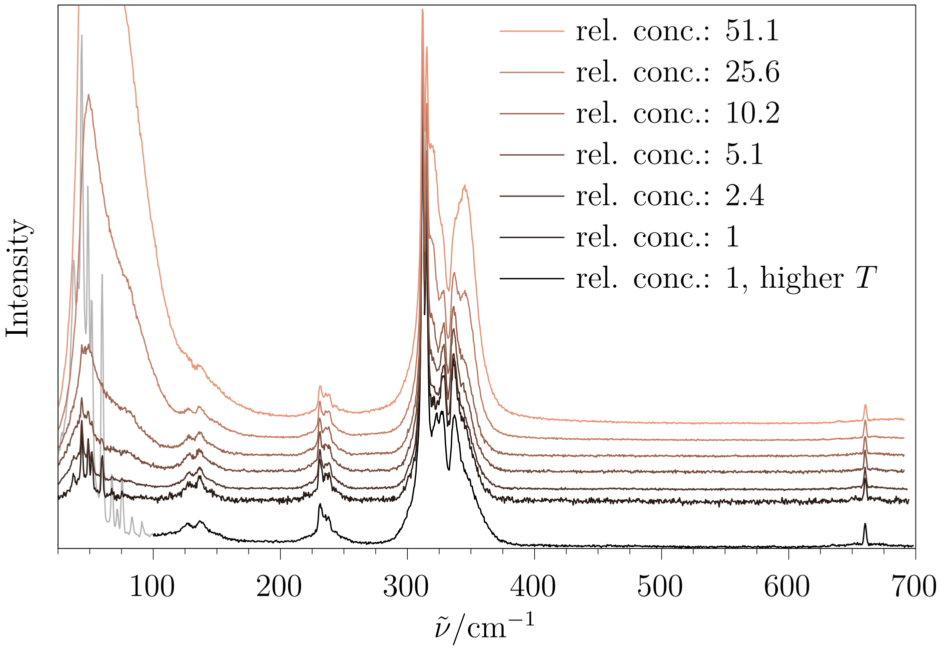

Fig. S1: Raman jet spectra of methyl methanoate at identical conditions except for various substance concentrations, scaled to the signal at 312 cm^{-1}. The lowest trace was recorded with a reduced distance to the nozzle (0.5 mm). The relative concentrations are comparable to those in Fig. 3 in the main text and Fig. S2. Below 100 cm^{-1} unusually large signals from air impurity are drawn greyed out in order to enhance readability.

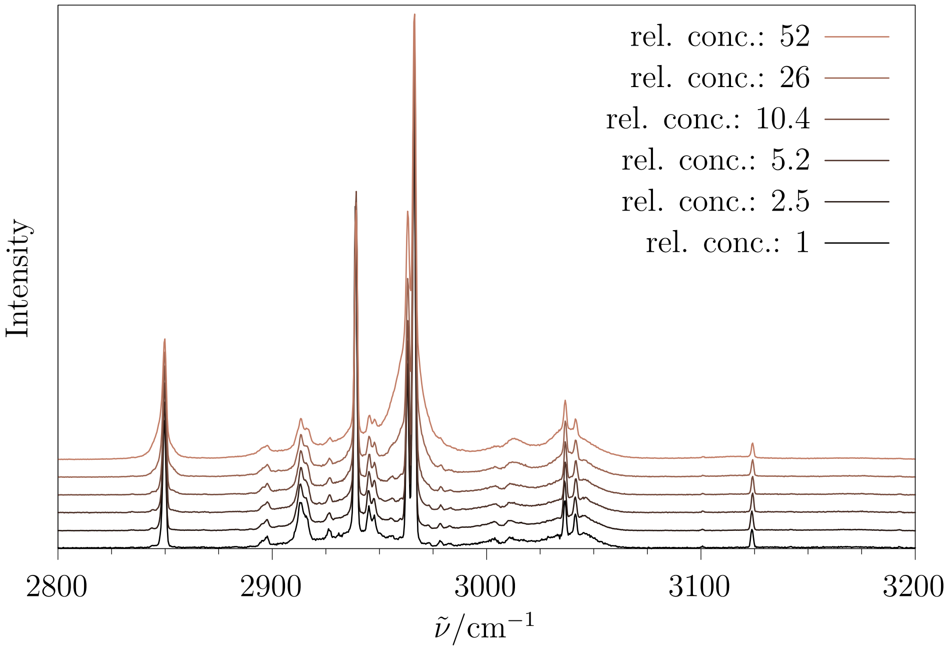

Fig. S2: Raman jet spectra of methyl methanoate at identical conditions except for various substance concentrations, scaled to the signal at 2966 cm^{-1}. The relative concentrations are comparable to those in Fig. 3 in the main text and Fig. S1.

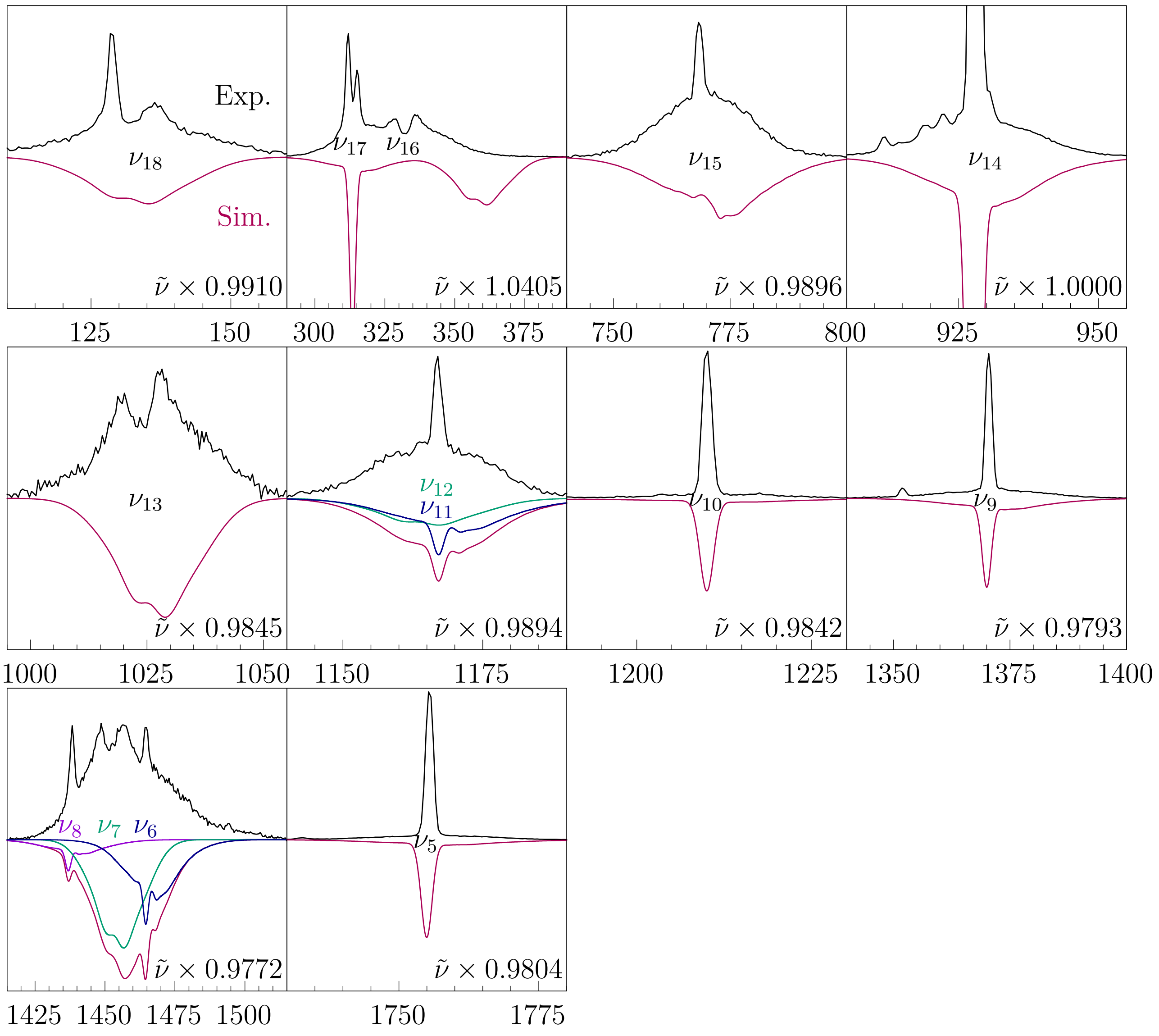

Fig. S3: Near-baseline sections of the jet spectrum of methyl methanoate (black trace) and rovibrational simulation (violet trace) for $T = 30\,\mathrm{K}$ and $\sigma = 1.0\,\mathrm{cm}^{-1}$. Calculated wavenumbers are scaled by the factors provided in the bottom row and calculated intensities are scaled to match the integrated experimental intensity, similarly to Fig. 5 in the main text.

The complex structure at around $1450\,\mathrm{cm}^{-1}$ deserves special discussion. At first glance, the experimental spectrum suggests that there are four vibrational transitions in this range. The simulation reveals that the two broad features in the middle are maxima of rotational branches of ν_7, and the two sharper signals are the Q^0 branches of ν_6 and ν_8. Of all fundamentals the simulation of ν_{15} matches the experimental spectrum the least. This is because the calculation predicts a'^2_{15} to be close to zero, and γ'^2_{15} to be almost exclusively of R_2 type: $\alpha_{xx} \approx -\alpha_{yy}$, $\alpha_{zz} \approx 0$ (see section 4). Therefore the Q^0-branch ($\Delta J = \Delta K = 0$) is predicted to be absent.

1.2 Methyl Butanoate

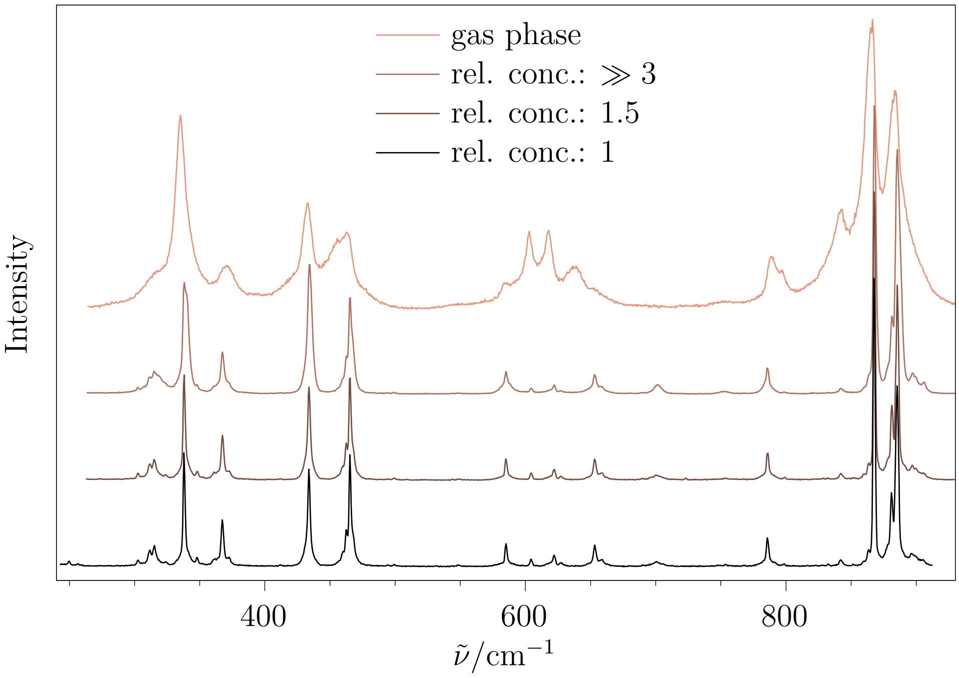

Fig. S4: Raman spectra of methyl butanoate. Top trace: gas phase. Lower three traces: jet spectra with identical conditions, except for varying ester concentration. The relative concentrations are comparable to those in Fig. 10 in the main text. Note the change of relative intensity of the signals at 434 and 465 cm^{-1} between the second and the lowest two traces due to clustering effects. No difference between spectra with relative concentration of 1 and 1.5 observable.

2 Additional Computational Results

2.1 Methyl Methanoate

Tab. S3: Effects of correction terms on calculated intensities, illustrated on the example of methyl methanoate, calculated for B3LYP/def2-QZVPP. a' and γ' (in atomic units, not squared) as obtained from the calculation with Turbomole. I_{raw} is the intensity for $0\,\mathrm{K}$ and an excitation wavelength of $532.27\,\mathrm{nm}$. C are the correction factors when only the solid angle of observation (θ, ϕ), or the dependence of the detection sensitivity of the monochromator on the polarisation of scattered photons (MC), or the vibrational temperature (T_{vib}) is taken into account. For comparison, the fully corrected intensities I_{full} are provided as well. It must be noted that the correction terms are not strictly multiplicative, because both $C_{\theta,\phi}$ and C_{MC} change the ratio of a'^2 and γ'^2. Also, the absolute value of I_{full} is meaningless because only relative intensities can be obtained from our spectra.

Mode	$\tilde{\nu}$/ cm^{-1}	a'/ a.u.	γ'/ a.u.	I_{raw}/ $10^{-35}\,\mathrm{m}^2/\mathrm{sr}$	$C_{\theta,\phi}$	C_{MC}	$C_{T_{\mathrm{vib}}}$	I_{full}/ $10^{-35}\,\mathrm{m}^2$
ν_{18}	133	0	0.014	1.014	1.033	0.857	1.263	1.051
ν_{17}	301	0.011	0.025	3.176	1.016	0.933	1.049	3.044
ν_{16}	344	0	0.048	4.603	1.033	0.854	1.034	4.085
ν_{15}	777	0.002	0.044	1.654	1.032	0.847	1.001	1.444
ν_{14}	928	0.034	0.069	8.221	1.013	0.937	1.000	7.794
ν_{13}	1042	0	0.036	0.766	1.033	0.834	1.000	0.659
ν_{12}	1176	0	0.036	0.660	1.033	0.828	1	0.564
ν_{11}	1180	−0.005	0.042	0.979	1.030	0.842	1	0.848
ν_{10}	1229	0.013	0.019	0.680	1.009	0.956	1	0.655
ν_9	1399	−0.018	0.057	2.252	1.020	0.892	1	2.044
ν_8	1470	−0.005	0.032	0.441	1.029	0.842	1	0.381
ν_7	1487	0	0.082	2.558	1.033	0.818	1	2.159
ν_6	1499	−0.006	0.063	1.583	1.031	0.827	1	1.347
ν_5	1790	−0.032	0.065	3.293	1.013	0.928	1	3.090
ν_4	3033	0.097	0.289	20.446	1.019	0.877	1	18.222
ν_3	3052	0.163	0.049	24.343	1.001	0.997	1	24.290
ν_2	3123	0	0.212	6.087	1.033	0.782	1	4.910
ν_1	3158	0.031	0.244	8.704	1.030	0.801	1	7.161

Tab. S4: Experimental and theoretical fundamental wavenumbers of methyl methanoate. B=B3LYP, P=PBE0, d=def2-QZVPP, dD=def2-QZVPPD, a=aug-cc-pVQZ. For vibrations without a sharp Q branch the average of the wavenumbers of the rotational maxima is given.

Mode	$\tilde{\nu}/\mathrm{cm}^{-1}$	B/d	B/dD	B/a	P/d	P/a
ν_{18}	132 ± 4	133	113	121	131	119
ν_{17}	312, 315	301	301	301	304	303
ν_{16}	332 ± 4	344	344	343	351	349
ν_{15}	769	777	777	776	786	786
ν_{14}	928	928	927	928	969	969
ν_{13}	1024 ± 4	1042	1041	1040	1051	1049
ν_{12}	1167	1176	1175	1175	1178	1177
ν_{11}	1167	1180	1179	1179	1193	1193
ν_{10}	1210	1229	1229	1229	1255	1255
ν_{9}	1370	1399	1398	1398	1399	1398
ν_{8}	1438	1470	1469	1470	1467	1466
ν_{7}	1452 ± 4	1487	1486	1487	1480	1480
ν_{6}	1465	1499	1497	1498	1493	1492
ν_{5}	1755	1790	1788	1789	1829	1827

Tab. S5: Calculated a' and γ'/a.u. of methyl methanoate. B=B3LYP, P=PBE0, d=def2-QZVPP, dD=def2-QZVPPD, a=aug-cc-pVQZ.

	B/d		B/dD		B/a		P/d		P/a	
Mode	a'	γ'	a'	γ'	a'	γ'	a'	γ'	a'	γ'
ν_{18}	0	0.014	0	0.011	0	0.011	0	0.014	0	0.011
ν_{17}	0.011	0.025	0.012	0.026	0.012	0.026	−0.010	0.024	−0.012	0.024
ν_{16}	0	0.048	0	0.045	0	0.045	0	0.047	0	0.044
ν_{15}	0.002	0.044	−0.002	0.039	0.002	0.039	0.002	0.042	0.002	0.037
ν_{14}	0.034	0.069	0.036	0.066	0.036	0.066	0.032	0.064	−0.034	0.061
ν_{13}	0	0.036	0	0.036	0	0.036	0	0.035	0	0.035
ν_{12}	0	0.036	0	0.027	0	0.027	0	0.033	0	0.024
ν_{11}	−0.005	0.042	−0.006	0.039	−0.006	0.039	−0.000	0.038	0.002	0.033
ν_{10}	0.013	0.019	0.013	0.019	0.013	0.020	0.014	0.023	−0.014	0.026
ν_{9}	−0.018	0.057	−0.017	0.051	−0.017	0.051	0.018	0.055	−0.017	0.050
ν_{8}	−0.005	0.031	0.004	0.029	−0.004	0.029	0.005	0.032	−0.005	0.030
ν_{7}	0	0.082	0	0.074	0	0.074	0	0.080	0	0.073
ν_{6}	−0.006	0.063	−0.006	0.056	0.006	0.056	0.006	0.062	0.007	0.056
ν_{5}	−0.032	0.065	0.039	0.068	0.039	0.068	−0.032	0.062	−0.038	0.064
ν_{4}	0.097	0.289	0.097	0.285	−0.098	0.286	−0.086	0.280	0.086	0.276
ν_{3}	0.163	0.049	−0.169	0.051	−0.169	0.051	0.168	0.047	−0.174	0.052
ν_{2}	0	0.212	0	0.209	0	0.210	0	0.208	0	0.205
ν_{1}	0.031	0.244	0.031	0.239	0.030	0.239	0.029	0.240	−0.029	0.236

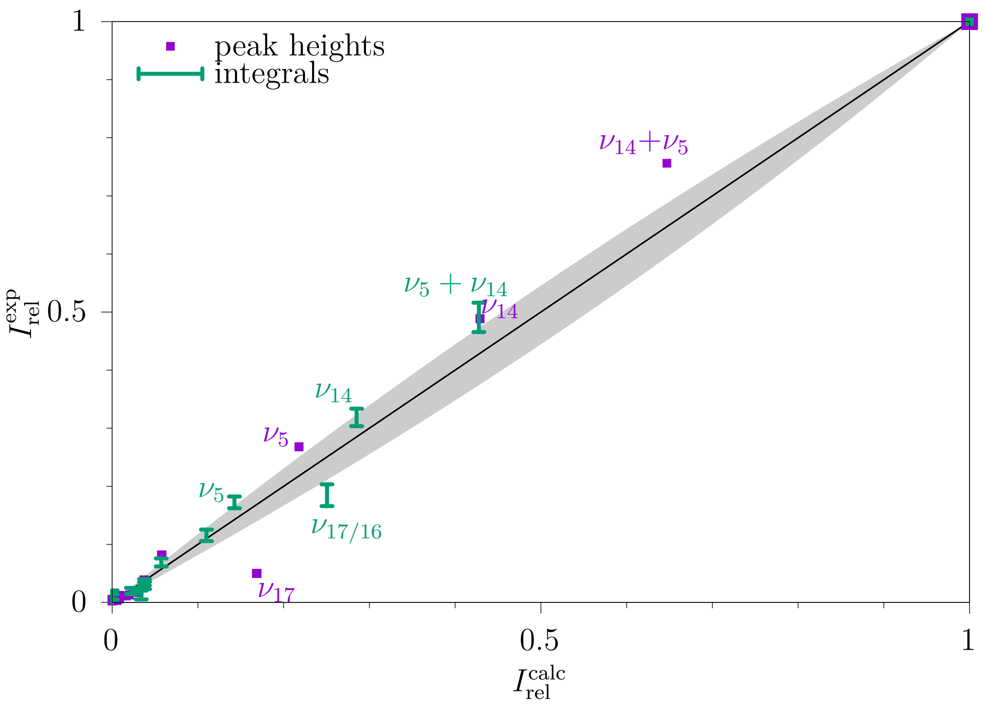

Fig. S5: Ratio of calculated (B3LYP/def2-QZVPPD) and experimental intensities for methyl methanoate. Violet points: peak height method. Green error bars: integral method; both normalised to give 1 in sum. Selected strong fundamentals and sums of fundamentals are labeled to exemplify that the deviations between experiment and theory are often correlated among the two methods. The area that would be covered by an anharmonicity effect of up to 20 % is shaded in grey. The agreement is typically better than for the less diffuse basis set in Fig. 8 in the main text.

2.2 Methyl Butanoate

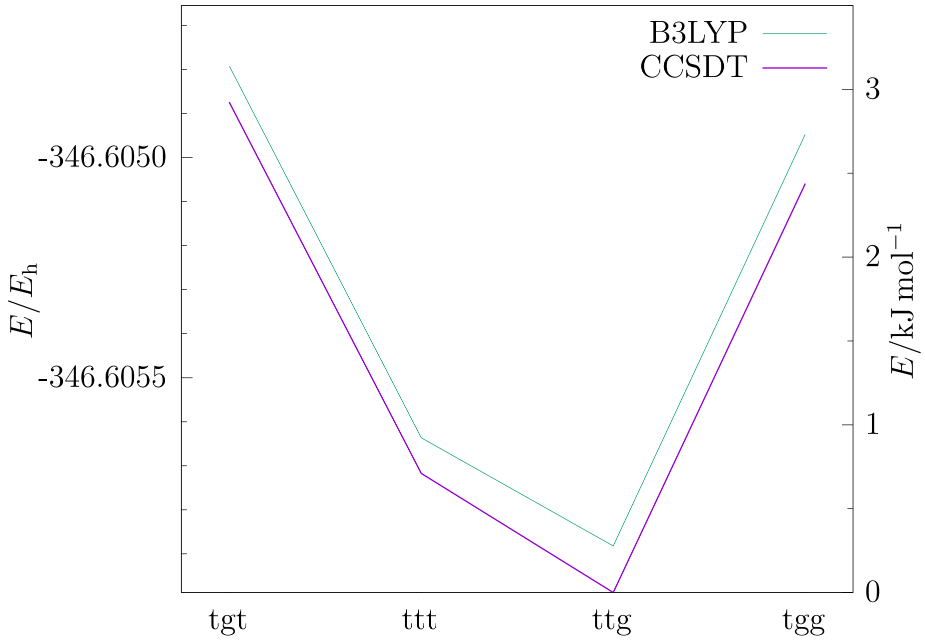

Fig. S6: Absolute single point electronic energies of the conformers of methyl butanoate on RIJK-CCSD(F12*)(T*)/cc-pVTZ-F12 level (Turbomole), calculated for structures optimised on different levels of theory. Green trace: optimised on B3LYP/def2-QZVPP level (Turbomole). Violet trace: Reference calculation, optimised on DF-CCSD(T)-F12A/cc-pVDZ-F12 level[S2,S3] (Molpro[S4–S6]). The absolute deviation is <0.28 kJ mol^{-1}, the deviation of relative energies is <0.07 kJ mol^{-1}. This suggests that the B3LYP structures are sufficiently accurate that their electronic energies can be replaced by CCSD(T) energies at least for the optimised conformations.

Tab. S6: Wavenumbers $\tilde{\nu}/\mathrm{cm}^{-1}$, (fully corrected) Raman cross sections $I/10^{-35}\,\mathrm{m}^2$ and symmetry character Γ of the four conformers of methyl butanoate, calculated on B3LYP/def2-QZVPP level, excluding the C−H stretching region. Those modes of ttt that resemble a vibration in methyl methanoate are labeled with the respective methanoate mode number. acc. = accordeon stretch.

tgt		ttt (C_s)				ttg		tgg	
$\tilde{\nu}$	I	$\tilde{\nu}$	I	Γ	Mode	$\tilde{\nu}$	I	$\tilde{\nu}$	I
20	116.7	34	9.5	a''		34	37.5	30	64.5
86	1.5	82	0.8	a''		103	6.3	86	7.4
117	2.7	139	1.4	a''	ν_{18}	140	0.9	138	1.4
143	0.7	149	0.5	a'		171	0.3	152	0.8
242	0.5	176	0.0	a''	ν_{16}	191	0.6	206	0.5
245	0.0	246	0.1	a''		256	0.4	250	0.5
288	1.1	309	2.5	a'	ν_{17}	310	0.6	309	2.9
327	5.9	340	6.1	a'	acc.	366	3.4	390	0.9
430	1.0	435	6.8	a'		467	5.8	450	1.1
654	5.5	588	0.3	a''		593	1.6	610	7.0
757	0.1	705	0.9	a'	ν_{15}	660	1.6	702	0.4
768	3.4	755	0.1	a''		794	1.4	808	1.9
857	3.7	890	0.2	a''		876	7.0	850	7.1
902	4.4	894	7.9	a'	(ν_{14})	891	3.6	879	4.5
914	1.0	912	0.2	a'		909	0.7	919	0.8
1023	4.3	1019	3.9	a'	(ν_{14})	1010	2.5	1008	2.0
1046	1.3	1051	3.1	a'		1065	1.6	1059	2.3
1108	0.9	1129	0.2	a''		1100	0.9	1101	0.7
1116	5.3	1133	3.5	a'		1128	2.0	1116	0.8
1177	0.5	1177	0.5	a''	ν_{12}	1177	0.5	1177	0.6
1210	0.9	1195	0.4	a'	ν_{11}	1201	0.8	1210	1.1
1229	0.3	1217	0.5	a'	ν_{10}	1220	0.3	1226	1.3
1281	0.2	1257	0.2	a''		1255	1.3	1280	1.4
1319	0.5	1330	2.4	a''		1302	1.3	1292	0.5
1340	1.9	1336	0.0	a'		1381	0.1	1364	1.4
1381	0.9	1406	0.1	a'		1393	0.7	1384	0.2
1417	0.0	1418	0.0	a'		1417	0.1	1423	0.0
1472	0.6	1462	2.6	a'		1463	2.7	1474	0.4
1486	4.2	1475	0.3	a'	ν_8	1474	0.3	1477	2.5
1486	1.3	1486	2.4	a''	ν_7	1486	2.3	1486	2.3
1497	2.4	1494	2.7	a'		1488	3.2	1493	3.4
1500	1.4	1501	1.8	a'	ν_6	1500	1.5	1500	1.4
1500	2.2	1502	1.9	a''		1502	1.0	1504	1.2
1512	0.2	1512	0.2	a'		1510	1.0	1505	0.3
1785	2.8	1788	2.1	a'	ν_5	1786	2.2	1787	2.4

Tab. S7: Wavenumbers $\tilde{\nu}$ and relative intensities I of methyl butanoate. (exp) experimental values from the argon enriched spectrum (trace e of Fig. 11), (B/d) calculated values on B3LYP/def2-QZVPP level. Intensities were separately normalised for each conformer to give 100 in sum. Reference wavenumbers from solid state, where both conformations appear to coexist[S1]. [a] Increased integration uncertainty due to partial overlap with neighbouring signal (see Fig. 12).
Calculations with diffuse basis sets def2-QZVPPD and aug-cc-pVQZ gave imaginary frequencies for all four conformers and are therefore not reported. The double-harmonic intensity calculation is in good agreement with the experiment for most of the signals, except for the two signals of ttg at (368 and 868) $\mathrm{cm^{-1}}$. Only if a favourable anharmonicity effect of up to 20 % is assumed, these two signals fall into the experimental uncertainty margin. As for methyl methanoate (Tab. 2), the intensity of the lowest tabulated ttg fundamental is overestimated by theory.

Conformer	$\tilde{\nu}_{\mathrm{exp}}/\mathrm{cm^{-1}}$	I_{exp}	$\tilde{\nu}_{\mathrm{B/d}}/\mathrm{cm^{-1}}$	$I_{\mathrm{B/d}}$	$\tilde{\nu}_{\mathrm{solid}}/\mathrm{cm^{-1}}$ [S1]
ttt	338	44.9 ± 5.1	340	44.2	344
	434	49.5 ± 4.4	435	49.2	436
	701	5.7 ± 2.4	705	6.6	705
ttg	368	10.8 ± 1.9	366	16.4	375
	465	26.9 ± 3.2	467	27.8	469
	585	5.2 ± 1.3	593	7.7	591
	653	6.5 ± 1.3	660	7.6	650
	786	6.6 ± 1.3	794	6.8	786
	868	44.1 ± 5.1^{a}	876	33.7	869

3 Instrumental Details

3.1 Characteristics of the CCD Chip

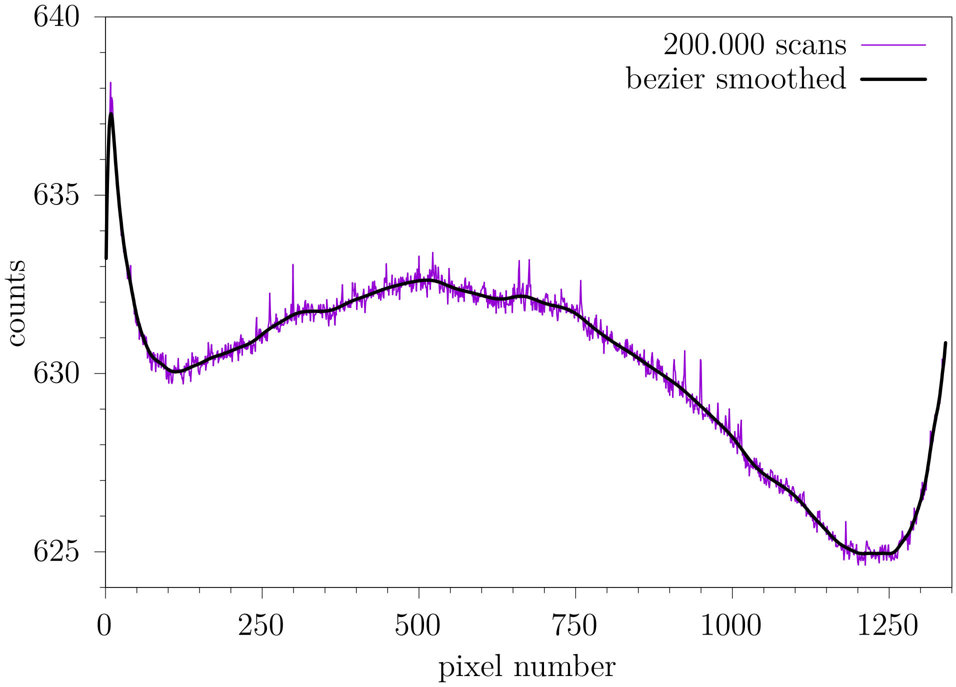

Fig. S7: Readout offset of the CCD camera obtained by averaging 200.000 readouts without external photons (closed shutter). The bezier smoothed curve is used for baseline correction of each spectrum. Note that the distortion of the baseline due to the readout offset of the chip (±7 counts) is smaller than the noise level of the best spectra in this work (±20 counts).

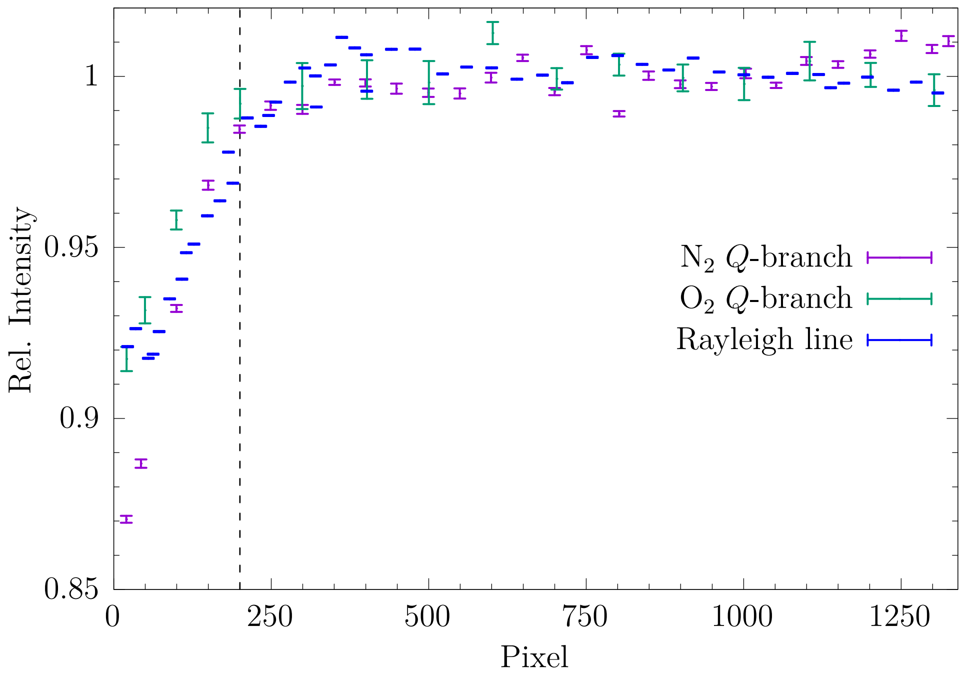

Fig. S8: The illumination of the CCD chip was tested by recording the Q-branch of N_2 and O_2 from air expansion and the Rayleigh scattering of residual gas on different positions on the CCD camera, which was achieved by changing the central wavelength of the monochromator. Relative intensities are normalised such that the mean intensity >200 cm^{-1} is 1. Nozzle distance 1 mm, exposure time 0.1 s, averaged over 100 scans for N_2 and O_2. For the Rayleigh line no expansion, exposure time 0.05 s, averaged over 100 scans. The intensity drop-off in the first 200 pixels indicates a shadowing effect which distorts Raman scattering intensities in this part of the spectrum by up to 15 %

3.2 Integration Over the Solid Angle of Observation

The scattering cross section $\sigma'_k(\theta, \phi)$ of a vibration k is dependent on the angle of observation where θ is the polar angle relative to the direction of propagation of the laser beam and ϕ is the azimuthal angle relative to the scattering plane.[S7] A scheme showing the scattering situation is shown in Fig. S9. In previous work of our group intensities were approximated by calculating σ' only for $\theta = 90°$ and $\phi = 0°$. Here the integration over the solid angle $\mathrm{d}\Omega = \sin\theta \mathrm{d}\theta \mathrm{d}\phi$ will be performed. To our knowledge, this is usually neglected.

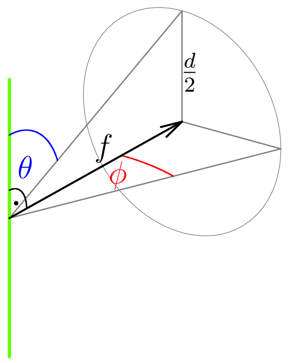

Fig. S9: Scheme of the photon collection geometry.

In general, σ'_k can be separated into a part V that is dependent on the wavenumber of the vibration $\tilde{\nu}_k$ containing the laser wavenumber and the vibrational temperature; and

into a part A' that is dependent on the angles of observation containing the isotropic and anisotropic transition polarisability invariants a_k^2 and γ_k^2 (Eq. 1). The prime $'$ symbolises the dependence on the angle of observation and will be dropped after integration.

$$\sigma'_k = V(\tilde{\nu}_k) \cdot A'(\theta, \phi) \tag{1}$$

$$\sigma_k = \int \sigma'_k \mathrm{d}\Omega \tag{2}$$

$$A = \int A'(\theta, \phi) \mathrm{d}\Omega \tag{3}$$

In fact, there are four cases to consider: Where the incident laser polarisation is orthogonal ($\perp^{\mathrm{i}}$) or parallel ($\|^{\mathrm{i}}$) to the scattering plane and where the scattered light is polarised either orthogonal ($\perp^{\mathrm{s}}$) or parallel ($\|^{\mathrm{s}}$) to the scattering plane. In our case, with no analyser in the beam, always both states of polarisation of the scattered light are recorded. The angular dependent part for these four cases is given in Eq. 4 to 7.[S7]. Eq. 7 is not given explicitely in Ref.[S7], but can readily be obtained from Eq. 5 by introducing a phase shift of $\pi/2$ in ϕ.

$$A'_k(\theta, \phi, \perp^{\mathrm{i}}, \perp^{\mathrm{s}}) = \frac{1}{45}(45a_k^2 + 4\gamma_k^2 - (45a_k^2 + \gamma_k^2)\sin^2\phi) \tag{4}$$

$$A'_k(\theta, \phi, \perp^{\mathrm{i}}, \|^{\mathrm{s}}) = \frac{1}{45}(3\gamma_k^2 + (45a_k^2 + \gamma_k^2)\cos^2\theta\sin^2\phi) \tag{5}$$

$$A'_k(\theta, \phi, \|^{\mathrm{i}}, \perp^{\mathrm{s}}) = \frac{1}{45}(45a_k^2 + 4\gamma_k^2 - (45a_k^2 + \gamma_k^2)\cos^2\phi) \tag{6}$$

$$A'_k(\theta, \phi, \|^{\mathrm{i}}, \|^{\mathrm{s}}) = \frac{1}{45}(3\gamma_k^2 + (45a_k^2 + \gamma_k^2)\cos^2\theta\cos^2\phi) \tag{7}$$

The camera objective that collects the scattered light has a collimation diameter of $d = 40\,\mathrm{mm}$ when the aperture is fully open and a focal length of $f = 50\,\mathrm{mm}$. Therefore light is collected at an angle of $\arctan(F) = \pm 21.8°$ with $F = d/2f$. Because integration is performed over a spherical cap, the integration interval of θ will be a function of ϕ. Additionally, due to the symmetry of the functions and the integrated surface, it is

sufficient to integrate over a quarter of the cap and multiply the result by 4. Doing so avoids ambiguities for angles >90°. Hence, the integral can be written as

$$A = 4 \int_{\phi=0}^{\arctan(F)} \int_{\theta_{\min}(\phi)}^{\pi/2} A' \sin\theta \mathrm{d}\theta \mathrm{d}\phi \tag{8}$$

From simple geometric considerations it follows that

$$\theta_{\min}(\phi) = \arctan\left(\frac{1}{\sqrt{F^2 - \tan^2\phi}}\right) \tag{9}$$

As can be seen from Eq. 4 to 7 there is always a constant term and an angular-dependent term involved. Unfortunately, we are not aware of analytical solutions for these integrals. Numerical double integration gives the following results:

$$4 \int_{\phi=0}^{\arctan(F)} \int_{\theta_{\min}(\phi)}^{\pi/2} \sin\theta \mathrm{d}\theta \mathrm{d}\phi = 0.45726316 \tag{10}$$

$$4 \int_{\phi=0}^{\arctan(F)} \int_{\theta_{\min}(\phi)}^{\pi/2} \sin^2\phi \sin\theta \mathrm{d}\theta \mathrm{d}\phi = 0.01664138 \tag{11}$$

$$4 \int_{\phi=0}^{\arctan(F)} \int_{\theta_{\min}(\phi)}^{\pi/2} \cos^2\theta \sin^2\phi \sin\theta \mathrm{d}\theta \mathrm{d}\phi = 0.00041443 \tag{12}$$

$$4 \int_{\phi=0}^{\arctan(F)} \int_{\theta_{\min}(\phi)}^{\pi/2} \cos^2\phi \sin\theta \mathrm{d}\theta \mathrm{d}\phi = 0.44062179 \tag{13}$$

$$4 \int_{\phi=0}^{\arctan(F)} \int_{\theta_{\min}(\phi)}^{\pi/2} \cos^2\theta \cos^2\phi \sin\theta \mathrm{d}\theta \mathrm{d}\phi = 0.01583374 \tag{14}$$

Since we are only considering relative intensities, the raw values of the integrals may be scaled arbitrarily. Here we chose to scale them such that for $A(\perp^{\mathrm{i}}, \perp^{\mathrm{s}})$ the term $45a_k^2$ is recovered:

$$A(\perp^\mathrm{i}, \perp^\mathrm{s}) = \frac{1}{45}(45a_k^2 + 4.113304\gamma_k^2) \tag{15}$$

$$A(\perp^\mathrm{i}, \|^\mathrm{s}) = \frac{1}{45}(0.042325a_k^2 + 3.114244\gamma_k^2) \tag{16}$$

$$A(\|^\mathrm{i}, \perp^\mathrm{s}) = \frac{1}{45}(1.699557a_k^2 + 3.151072\gamma_k^2) \tag{17}$$

$$A(\|^\mathrm{i}, \|^\mathrm{s}) = \frac{1}{45}(1.617074a_k^2 + 3.149239\gamma_k^2) \tag{18}$$

Compared to the description without integration, for measurements with 90° laser polarisation ($\perp^\mathrm{i}$) there is only a small increase of the contribution from anisotropy, but for measurements with 0° laser polarisation ($\|^\mathrm{i}$) the effects are much larger.

For completeness, the general trend of the scaled prefactors for $F = [0 : 2]$ is shown in Fig. S10. For $F \to \infty$, which corresponds to light collection over a half sphere, the integration over a coupled interval becomes equal to independent double integration over $[0 : \pi/2]$, which is solvable analytically. At this limit the dependence on the incident laser polarisation is lost: $A_k^\infty(\perp^\mathrm{s}) = 45a_k^2 + 7\gamma_k^2$ and $A_k^\infty(\|^\mathrm{s}) = {}^1\!/_3(45a_k^2 + 19\gamma_k^2)$.

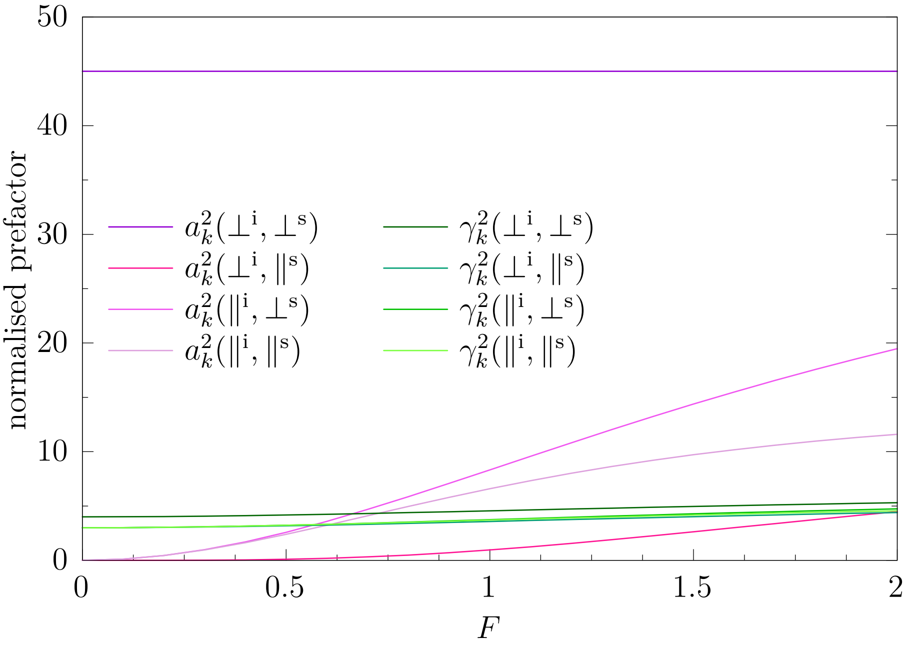

Fig. S10: Dependence of prefactors in Eq. 15 – 18 on F, normalised to $45a_k^2$ in $A_k(\perp^\mathrm{i}, \perp^\mathrm{s})$.

4 Explicit Rotational Contours

4.1 Symmetric Top

In this study, the molecules of interest resemble prolate symmetric top molecules, therefore the description of rotational broadening is derived from the true symmetric top case. The theoretical description is taken from Derek Long, The Raman Effect[S7].

For a prolate symmetric top molecule, each vibration is accompanied by a $\Delta J = 0$, ± 1 and ± 2 irrespective of its symmetry, but for ΔK either 0, ± 1 or ± 2 are allowed, depending on the symmetry character of the vibration. The intensity of such a single rovibrational transition is the product of the usual term from vibrational theory, the relative population of the initial rotational state, the Placzek-Teller-factor of the corresponding transition and the activity as a function of a^2 and γ^2. For convenience, the Placzek-Teller factors are shown in Tab. S8 and S9[S7].

Tab. S8: Placzek-Teller factors

ΔK \ ΔJ	-2	-1	0
-2	$\frac{(J+K-3)(J+K-2)(J+K-1)(J+K)}{4J(J-1)(2J-1)(2J+1)}$	$\frac{\left(J^2-(K-1)^2\right)(J+K-2)(J+K)}{2J(J-1)(J+1)(2J+1)}$	$\frac{3\left(J^2-(K-1)^2\right)\left((J+1)^2-(K-1)^2\right)}{2J(J+1)(2J-1)(2J+3)}$
-1	$\frac{\left((J-1)^2-(K-1)^2\right)(J+K-1)(J+K)}{J(J-1)(2J-1)(2J+1)}$	$\frac{(J-2K+1)^2(J+K-1)(J+K)}{2J(J-1)(J+1)(2J+1)}$	$\frac{3(2K-1)^2(J+K)(K-K+1)}{2J(J+1)(2J-1)(2J+3)}$
0	$\frac{3\left((J-1)^2-K^2\right)\left(J^2-K^2\right)}{2J(J-1)(2J-1)(2J+1)}$	$\frac{3K^2(J^2-K^2)}{J(J-1)(J+1)(2J+1)}$	$\frac{\left(J(J+1)-3K^2\right)^2}{J(J+1)(2J-1)(2J+3)}$
$+1$	$\frac{\left((J-1)^2-(K+1)^2\right)((J-K-1)(J-K))}{J(J-1)(2J-1)(2J+1)}$	$\frac{(J+2K+1)^2(J-K-1)(J-K)}{2J(J-1)(J+1)(2J+1)}$	$\frac{3(2K+1)^2(J-K)(J+K+1)}{2J(J+1)(2J-1)(2J+3)}$
$+2$	$\frac{(J-K-3)(J-K-2)(J-K-1)(J-K)}{4J(J-1)(2J-1)(2J+1)}$	$\frac{\left(J^2-(K+1)^2\right)(J-K-2)(J-K)}{2J(J-1)(J+1)(2J+1)}$	$\frac{3\left(J^2-(K+1)^2\right)\left((J+1)^2-(K+1)^2\right)}{2J(J+1)(2J-1)(2J+3)}$

The Placzek-Teller factors have the property that for any signed ΔK the sum over all ΔJ is equal 1, i.e. in the symmetric top case for a vibration with $\Delta K = \pm 1$ or ± 2 the sum over all Placzek-Teller factors is 2. In our understanding, this would double the total intensity of such a vibration if rotation is explicitly included. In order to preserve the overall intensity our pragmatical approach is to divide the intensity of each line associated with a $\Delta K \neq 0$ by 2, except for all transitions from states with $K = 0$ and those with $\Delta K = 2$ from $K = 1$.

Tab. S9: Placzek-Teller factors (continuation)

ΔK \ ΔJ	$+1$	$+2$
-2	$\frac{\big((J+1)^2-(K-1)^2\big)(J-K+1)(J-K+3}{2J(J+1)(J+2)(2J+1)}$	$\frac{(J-K+1)(J-K+2)(J-K+3)(J-K+4)}{4(J+1)(J+2)(2J+1)(2J+3)}$
-1	$\frac{(J+2K)^2(J-K+1)(J-K+2)}{2J(J+1)(J+2)(2J+1)}$	$\frac{\big((J+1)^2-K^2\big)(J-K+2)(J-K+3)}{(J+1)(J+2)(2J+1)(2J+3)}$
0	$\frac{3K^2\big((J+1)^2-K^2\big)}{J(J+1)(J+2)(2J+1)}$	$\frac{3\big((J+1)^2-K^2\big)\big((J+2)^2-K^2\big)}{2(J+1)(J+2)(2J+1)(2J+3)}$
1	$\frac{(J-2K)^2(J+K+1)(J+K+2)}{2J(J+1)(J+2)(2J+1)}$	$\frac{\big((J+1)^2-K^2\big)(J+K+2)(J+K+3)}{(J+1)(J+2)(2J+1)(2J+3)}$
2	$\frac{\big((J+1)^2-(K+1)^2\big)(J+K+1)(J+K+3)}{2J(J+1)(J+2)(2J+1)}$	$\frac{(J+K+1)(J+K+2)(J+K+3)(J+K+4)}{4(J+1)(J+2)(2J+1)(2J+3)}$

The isotropic and anisotropic invariants a^2 and γ^2 are calculated by Eq. 19 and 20.

$$a^2 = \frac{1}{9}\left(\alpha_{xx} + \alpha_{yy} + \alpha_{zz}\right)^2 \tag{19}$$

$$\gamma^2 = \frac{1}{2}\left((\alpha_{xx} - \alpha_{yy})^2 + (\alpha_{xx} - \alpha_{zz})^2 + (\alpha_{yy} - \alpha_{zz})^2\right) + 3\left(\alpha_{xy}^2 + \alpha_{xz}^2 + \alpha_{yz}^2\right) \tag{20}$$

For each case, some components of the transition polarisability are zero or are equal to each other (Tab. S10). In general, a^2 contributes solely to the Q^0-branch, while γ^2 is responsible for all 25 possible rotational branches.

Tab. S10: Value of components of transition polarisability for vibrations of different symmetry for symmetric top molecules, and the associated selection rule for ΔK(Herzberg, p. 441[S8]).

Type	ΔK	α_{xx}	α_{yy}	α_{zz}	α_{xy}	α_{xz}	α_{yz}
R_0	0	$\neq 0$	α_{xx}	$\neq 0$	—	—	—
R_1	± 1	—	—	—	—	$\neq 0$	$\neq 0$
R_2	± 2	$\neq 0$	$-\alpha_{xx}$	—	$\neq 0$	—	—

4.2 Separation of γ

For an asymmetric top molecule there are no symmetry restrictions on the selection rules, hence each vibration will show 25 branches for all possible combinations of ΔJ and ΔK. When describing a transition of an asymmetric top where all ΔK values are allowed, it is necessary to use a reduced γ for each ΔK such that $\gamma_0^2 + \gamma_1^2 + \gamma_2^2 = \gamma^2$ in order to

preserve the total intensity. The simplest approach could be to set $\gamma_0^2 = \gamma_1^2 = \gamma_2^2 = {}^1\!/\!_3\gamma^2$, but this would asymptotically give wrong results for a true symmetric top. Based on the symmetry properties shown in Tab. S10 a more sophisticated distribution may be achieved by assigning the separate terms in Eq. 20 to different types R_0, R_1, R_2:

$$\gamma^2 = \underbrace{\alpha_{zz}\left(\alpha_{zz} - \alpha_{xx} - \alpha_{yy}\right)}_{R_0} + \underbrace{\alpha_{xx}^2 + \alpha_{yy}^2 - \alpha_{xx}\alpha_{yy}}_{R_0+R_2} + \underbrace{3\alpha_{xy}^2}_{R_2} + \underbrace{3\left(\alpha_{xz}^2 + \alpha_{yz}^2\right)}_{R_1} \tag{21}$$

Because the second term in Eq. 21 will contribute to both R_0 and R_2, further separation is needed. In the literature on Raman intensities a term for the intensity ratio of ${}^{R_2}\!/\!_{R_0}$ was derived:[S9,S10]

$$\frac{R_2}{R_0} = \frac{\frac{1}{\sqrt{2}}(\alpha_{xx} - \alpha_{yy})}{\frac{1}{\sqrt{6}}(2\alpha_{zz} - \alpha_{xx} - \alpha_{yy})} \tag{22}$$

As a side note, this ratio resembles the inverse of Ray's κ that is used to describe how close a molecule is to a truly symmetric top:

$$\kappa = \frac{2B - A - C}{A - C} \tag{23}$$

By setting $R_0 + R_2 = 1$ the expressions in Eq. 24 may be obtained. By taking the absolute value of ${}^{R_2}\!/\!_{R_0}$ it is ensured that R_0 and R_2 both have values in $[0:1]$, and are exactly 0 or 1 in the true symmetric top case.

$$R_0 = \frac{1}{1 + \left|\frac{\sqrt{3}(\alpha_{xx} - \alpha_{yy})}{2\alpha_{zz} - \alpha_{xx} - \alpha_{yy}}\right|} \tag{24}$$

$$R_2 = 1 - R_0 \tag{25}$$

Hence, the resulting expressions for the three γ's may be formed:

$$\gamma_0^2 = \alpha_{zz}\left(\alpha_{zz} - \alpha_{xx} - \alpha_{yy}\right) + R_0\left(\alpha_{xx}^2 + \alpha_{yy}^2 - \alpha_{xx}\alpha_{yy}\right) \tag{26}$$

$$\gamma_1^2 = 3\left(\alpha_{xz}^2 + \alpha_{yz}^2\right) \tag{27}$$

$$\gamma_2^2 = R_2\left(\alpha_{xx}^2 + \alpha_{yy}^2 - \alpha_{xx}\alpha_{yy}\right) + 3\alpha_{xy}^2 \tag{28}$$

We do not claim that this separation is the only valid one, but it preserves the overall intensity and asymptotically gives correct results when applied to a truly symmetric top molecule.

4.3 Rotational Temperature

Excitations are calculated for states up to $J_{\max}$ and $K_{\max}$ for which the population relative to $J = K = 0$ is at least 0.01 at the chosen rotational temperature. The sum over all population numbers is set to 1.

4.4 Rotational Constants

For a symmetric top molecule two of the three rotational constants A, B, C are equal: for prolate $A > B = C$, for oblate $A = B > C$.[S7] Asymmetric tops can be classified to resemble prolate or oblate symmetric tops by Ray's κ (Eq. 23), which is -1 for prolate and $+1$ for oblate.

In our approach we use the average of the two similar rotational constants as an effective B, depending on the sign of κ. (as is done in Herzberg, p.48, formulae (I,65) and (I,66)[S8])

5 Transformation of Raman Results from Turbomole

5.1 Obtaining Intensities Comparable to our Setup

For convenience, a short Python3 script is provided that takes the vibrational wavenumber $\tilde{\nu}/\mathrm{cm}^{-1}$, a and γ/a.u. from a Turbomole Raman calculation and transforms them to a scattering cross section that is comparable to the experimental intensities recorded at our setup.

```
def curry_int(ny, alpha, gamma):
        # from Turbomole calculation: ny in cm^-1, alpha and gamma in a.u.
        import numpy as np
        from numpy import (exp, sqrt, pi, array)

        # constants in SI
        h = 6.62607015e-34
        c = 2.99792458e8
        a0 = 5.29177210903e-11
        me = 9.1093837015e-31
        kB = 1.380649e-23
        u = 1.66053906660e-27
        # laser wavelength
        laser = 532.27e-9

        # alpha^2 and gamma^2 in SI
        alpha_sq = a0**4/me*alpha**2
        gamma_sq = a0**4/me*gamma**2

        # definition of polarisation correction function
        def curry_func(ny):
                return (
                1.7654
                +2.6970e-10*(ny-2000)**3
                +0.4316e-13*(ny-2000)**4
                -1.1285e-16*(ny-2000)**5
                -0.1278e-19*(ny-2000)**6
                +0.1926e-22*(ny-2000)**7
                +0.1029e-26*(ny-2000)**8
                -1.1527e-30*(ny-2000)**9
                )

        # prefactor as abbreviation
        fact = 2*pi**2*h/(45*c*laser)

        # temperature factor
        def exp_func(ny,T):
                return (
                1/(1-exp(-h*c*ny*100/(kB*T)))
                )

        # intensity is multiplied with average of 20 and 180 K
        def T_func(ny):
                return (
                (exp_func(ny,20) + exp_func(ny,180))/2
                )

        # return result for orthogonal incident laser polarisation
        return (
        fact*(1/laser-100*ny)**3
        /(100*ny)*T_func(ny)
        *(45*alpha_sq + 4.113304*gamma_sq
        + (0.042325*alpha_sq + 3.114244*gamma_sq)/curry_func(ny))
        )
```

5.2 Components of Transition Polarisability

Per default, Turbomole does not print the components of the transition polarisability $\alpha_{xx}, \alpha_{xy}...\alpha_{zz}$, but only the averages a and γ. Here we briefly describe how these can be calculated from the results of a typical Raman calculation.

Two quantities are needed: The cartesian derivatives of all six components of the electronic polarisability w.r.t. nuclear coordinates, and the mass-un-weighted normal modes.

The normal modes that are printed in `vib_normal_modes` are not usable because there translation and rotation are not projected out. Properly separated normal modes can be created with the tool `tm2molden` that comes along any Turbomole installation. The elements are sorted from left to right, from top to bottom, in the order $x_1, y_1, z_1, x_2...$ These normal modes are still mass-weighted, which has to be reversed. The reduced masses μ_k of each vibration k can be readily extracted from the file `control`, where the elements are sorted from left to right, from top to bottom, in ascending order from μ_1 to μ_n. The coordinates of a normal mode have then to be divided by $\sqrt{\mu_k \cdot \mathrm{u}/m_\mathrm{e}}$ where u is the atomic mass unit and m_e is the electron mass. At this stage it is convenient to reshape the coordinates of a normal mode to a column vector.

The derivatives of the components of the polarisability are printed in the file `control`, from left to right, from top to bottom, in ascending order $x_1, y_1, z_1, x_2...$ It is convenient to reshape the elements into a row vector. Now the α_{xx} of the normal mode k is simply the scalar product of the derivatives of the xx component and the normal mode k. The other five components may be obtained in a similar manner, with their unit being atomic units $({a_0}^2 {m_\mathrm{e}}^{-1/2})$.

References

(S1) Oakes, R. E.; Beattie, J. R.; Moss, B. W.; Bell, S. E. Conformations, vibrational frequencies and Raman intensities of short-chain fatty acid methyl esters using DFT with 6-31G(d) and Sadlej pVTZ basis sets. *J. Mol. Struct. THEOCHEM* **2002**, *586*, 91–110.

(S2) Adler, T. B.; Knizia, G.; Werner, H.-J. A simple and efficient CCSD(T)-F12 approximation. *J. Chem. Phys.* **2007**, *127*, 221106.

(S3) Győrffy, W.; Knizia, G.; Werner, H.-J. Analytical energy gradients for explicitly correlated wave functions. I. Explicitly correlated second-order Møller-Plesset perturbation theory. *J. Chem. Phys.* **2017**, *147*, 214101.

(S4) Werner, H.-J.; Knowles, P. J.; Knizia, G.; Manby, F. R.; Schütz, M. Molpro: a general-purpose quantum chemistry program package. *WIREs Comput Mol Sci* **2011**, *2*, 242–253.

(S5) Werner, H.-J.; Knowles, P. J.; Manby, F. R.; Black, J. A.; Doll, K.; Heßelmann, A.; Kats, D.; Köhn, A.; Korona, T.; Kreplin, D. A.; Ma, Q.; Miller, T. F.; Mitrushchenkov, A.; Peterson, K. A.; Polyak, I.; Rauhut, G.; Sibaev, M. The Molpro quantum chemistry package. *J. Chem. Phys.* **2020**, *152*, 144107.

(S6) Werner, H.-J.; Knowles, P. J.; Knizia, G.; Manby, F. R.; Schütz, M.; Celani, P.; Györffy, W.; Kats, D.; Korona, T.; Lindh, R.; Mitrushenkov, A.; Rauhut, G.; Shamasundar, K. R.; Adler, T. B.; Amos, R. D.; Bennie, S. J.; Bernhardsson, A.; Berning, A.; Cooper, D. L.; Deegan, M. J. O.; Dobbyn, A. J.; Eckert, F.; Goll, E.; Hampel, C.; Hesselmann, A.; Hetzer, G.; Hrenar, T.; Jansen, G.; Köppl, C.; Lee, S. J. R.; Liu, Y.; Lloyd, A. W.; Ma, Q.; Mata, R. A.; May, A. J.; McNicholas, S. J.; Meyer, W.; Miller III, T. F.; Mura, M. E.; Nicklass, A.; O'Neill, D. P.; Palmieri, P.; Peng, D.; Pflüger, K.; Pitzer, R.; Reiher, M.; Shiozaki, T.; Stoll, H.; Stone, A. J.; Tarroni, R.; Thorsteinsson, T.; Wang, M.; Welborn, M. MOLPRO, version 2019.2, a package of ab initio programs. see https://www.molpro.net.

(S7) Long, D. A. *The Raman effect : a unified treatment of the theory of Raman scattering by molecules*; Wiley: Chichester New York, 2002.

(S8) Herzberg, G. *Molecular Spectra and Molecular Structure*; D. Van Nostrand Company, Inc.: 120 Alexander St., Princeton, New Jersey, 1945; Vol. II. Infrared and Raman Spectra of Polyatomic Molecules.

(S9) Hills, G. W.; Jones, W. J. Raman spectra of asymmetric top molecules. Part 1.—The pure rotational spectrum of ethylene. *J. Chem. Soc., Faraday Trans. 2* **1975**, *71*, 812–826.

(S10) Hills, G.; Foster, R.; Jones, W. Raman spectra of asymmetric top molecules. *Mol. Phys.* **1977**, *33*, 1571–1588.
